# Arg72Pro Polymorphism of *TP53* Gene and the Risk of Skin Cancer: a Meta-Analysis

**DOI:** 10.1371/journal.pone.0079983

**Published:** 2013-11-08

**Authors:** Jun Ye, Xiao-Fen Li, Yong-Dong Wang, Ying Yuan

**Affiliations:** 1 Department of Dermatology, Sir Run Run Shaw Hospital, Zhejiang University School of Medicine, Hangzhou, China; 2 Department of Medical Oncology, the Second Affiliated Hospital, Zhejiang University School of Medicine, Hangzhou, China; 3 Key Laboratory of Cancer Prevention and Intervention of Ministry of Education, the Second Affiliated Hospital, Zhejiang University School of Medicine, Hangzhou, China; Ohio State University Medical Center, United States of America

## Abstract

**Background:**

*TP53* gene is one of the most important tumor suppressor genes. We undertook this meta-analysis to explore the association between *TP53* Arg72Pro polymorphism and the risk of skin cancer mainly in Caucasians.

**Methods:**

We searched PubMed for case-control studies published up to March 2013. Odds ratios (ORs) with 95% confidence intervals (CIs) were used to assess the strength of association.

**Results:**

A total of 5276 skin cancer cases and 5315 controls from 20 studies were included. Overall, no significant association between *TP53* Arg72Pro polymorphism and skin cancer was observed in all genetic contrast models (Pro/Pro versus Arg/Arg, Pro/Arg versus Arg/Arg, Pro/Pro + Pro/Arg versus Arg/Arg, Pro/Pro versus Arg/Arg + Pro/Arg, Pro allele versus Arg allele). Similar results were obtained in the stratified analysis by ethnicity and histological types of skin cancer, such as melanoma, squamous cell carcinoma and basal cell carcinoma. Power calculations indicated that some studies were underpowered. No publication bias was found by using the funnel plot and Egger's test.

**Conclusions:**

This meta-analysis indicated that *TP53* Arg72Pro polymorphism probably had little association with skin cancer susceptibility mainly in Caucasians. However, larger sample-size studies are required to verify the conclusion as low statistical powers.

## Introduction

According to epidemiology, skin cancer including melanoma and non-melanoma is the most common type of cancer in white populations [1]. Statistics show that the incidence of skin cancer has been increasing in Europe and the USA, especially melanoma, in the past two decades [2,3]. Skin cancer has several histological subtypes, including melanoma, squamous cell carcinoma (SCC) and basal cell carcinoma (BCC) [4]. Many studies indicate that ultraviolet (UV) exposure is a major risk factor of skin cancer development [5-7]. However, on the molecular level, the carcinogenic mechanism of UV has not been expounded yet. 

*TP53* gene is a tumor suppressor gene which can regulate cell cycle arrest, cell apoptosis and DNA repair [8]. Hence, it is called guardian of genome. Mutations of *TP53* gene are the most common genetic abnormality found in many kinds of human cancers, such as lung cancer, colon cancer, gastric cancer, skin cancer, et al [9]. Arg72Pro polymorphism of *TP53* gene is a G-C transversion at codon 72, resulting in an amino acid change from arginine (Arg) to proline (Pro) [10]. Studies have shown that *TP53* gene plays an important role in the cellular genome protection from UV exposure [11,12]. But the detailed molecular mechanism is unclear.

Many studies in recent years have investigated the association between *TP53* Arg72Pro polymorphism and the risk of skin cancer, but their results remain inconclusive. Thus, we performed this meta-analysis of all eligible case–control studies that have been published to help us for a better understanding of the influence of *TP53* Arg72Pro polymorphism.

## Methods

### Publication Search

We searched PubMed for publications up to March 2013, using the terms “TP53,” “polymorphism,” and “skin cancer.” The search was performed without any restrictions on language. Besides, we searched the reference lists of reviews and retrieved articles manually. When the same patient population appeared in several articles, we chose the largest sample size or the most recent one.

### Inclusion Criteria

The selected studies must have met the following major criteria: (1) well-designed case-control studies to evaluate *TP53* Arg72Pro polymorphism and the risk of skin cancer; (2) skin cancer was diagnosed by pathology; (3) containing useful genotype frequencies; and (4) the distribution of genotypes among controls were in Hardy-Weinberg equilibrium.

### Exclusion Criteria

The exclusion criteria included: (1) the genotype frequencies or number not presented; (2) animal studies, reviews, case reports, abstracts and family-based studies; (3) duplication of a previous publication.

### Data Extraction

Two investigators extracted information from eligible studies independently, according to the inclusion and exclusion criteria above. Disagreements were resolved by discussion or a third investigator. The following information was collected: first authors, publication year, ethnicity, characteristics of cases and controls (mean age, distribution of gender), histological type of cases, genotyping method, number of genotypes and total number of cases and controls. 

In the paper of Rizzato et al the non coding strand has been genotyped, so we inverted the genotypes in his paper.

### Statistical Analysis

The strength of the association between *TP53* Arg72Pro polymorphism and the risk of skin cancer was evaluated by pooled odds ratios (ORs) with 95% confidence intervals (CIs). The pooled ORs for dominant model (Arg/Arg + Pro/Arg versus Pro/Pro), recessive model (Arg/Arg versus Arg/Pro + Pro/Pro), codominant model (Arg/Arg versus Pro/Pro and Arg/Pro versus Pro/Pro) and the allele contrast (Pro allele versus Arg allele) were calculated, respectively. Stratified analyses were performed by ethnicity and histological type of skin cancer. The heterogeneity assumption was assessed by the Chi-square-based Q-test. If P<0.05 of the Q-test which indicated heterogeneity, the random-effects model was used to calculate the pooled ORs. Otherwise, the fixed-effects model was adopted. The Z test was applied to determine the pooled OR with the significance set at P<0.05. Potential publication bias was estimated by Begg’s funnel plot [13] and Egger’s test [14]. P>0.05 meant no significant publication bias. All above statistical analyses were performed with the STATA software, version 12.0 (StataCorp, College Station, TX, USA). Power analysis was performed using the Power and Sample Size Calculation (PS) program (http://biostat.mc.vanderbilt.edu/wiki/Main/PowerSampleSize) [15].

## Results

### Study Characteristics

A total of 165 papers were obtained by the publication search published until March 2013, among which twenty met the inclusion criteria [16-35] (Figure S1). The ultimate twenty studies were all in English, involving 5276 skin cancer cases and 5315 controls. The main characteristics were summarized in Table 1. 

**Table 1:** Characteristics of studies included in the meta-analysis.

First author	Year	Ethnicity	Country	Cases			Controls		
				Pro/Pro	Pro/Arg	Arg/Arg	Pro/Pro	Pro/Arg	Arg/Arg
Dokianakis	2000	Caucasian	Greece	3	5	19	6	41	12
Marshall	2000	Caucasian	England	3	18	34	6	39	39
Bastiaens	2001	Caucasian	The Netherlands	21	131	169	10	72	75
O'Connor	2001	Caucasian	Ireland	1	11	43	4	20	91
Cairey-Remonnay	2002	Caucasian	France	4	16	50	5	66	85
McGregor	2002	Caucasian	England	0	58	124	5	7	17
Gustafsson	2004	Caucasian	Sweden	5	19	30	3	31	62
de Oliveira	2004	Other	Brazil	0	0	16	2	9	16
Gwosdz	2006	Caucasian	Germany	7	24	18	13	66	114
Han	2006	Caucasian	USA	55	294	409	45	297	474
Pezeshki	2006	Asian	Iran	10	47	34	86	217	162
Stefanaki	2007	Caucasian	Greece	11	44	52	6	66	73
Bendesky	2007	Other	Mexico	25	94	122	18	94	126
Queille	2007	Caucasian	France	2	15	13	6	39	39
Li	2008	Caucasian	USA	40	300	465	56	350	432
Capasso	2010	Caucasian	Italy	30	87	123	23	122	139
Almquist	2011	Caucasian	USA	94	551	851	47	274	446
Rizzato	2011	Caucasian	Hungary, Romania, Slovakia	40	186	292	46	178	297
Leob	2012	Caucasian	USA	4	16	35	5	19	17
Pandish	2012	Asia	India	19	62	25	32	78	90
Melanomas									
Bastiaens	2001	Caucasian	The Netherlands	7	48	65	10	72	75
Gwosdz	2006	Caucasian	Germany	7	24	18	13	66	114
Han	2006	Caucasian	USA	15	82	104	45	297	474
Stefanaki	2007	Caucasian	Greece	11	44	52	6	66	73
Li	2008	Caucasian	USA	40	300	465	56	350	432
Capasso	2010	Caucasian	Italy	30	87	123	23	122	139
SCC									
Dokianakis	2000	Caucasian	Greece	0	1	2	6	41	12
Marshall	2000	Caucasian	England	2	14	18	6	39	39
Bastiaens	2001	Caucasian	The Netherlands	6	40	41	10	72	75
Cairey-Remonnay	2002	Caucasian	France	4	16	50	5	7	17
McGregor	2002	Caucasian	England	0	35	74	5	66	85
Gustafsson	2004	Caucasian	Sweden	5	19	30	3	31	62
Han	2006	Caucasian	USA	17	104	151	45	297	474
Bendesky	2007	Other	Mexico	3	21	18	126	94	18
Almquist	2011	Caucasian	USA	37	220	366	47	274	446
Leob	2012	Caucasian	USA	4	16	35	5	19	17
Pandish	2012	Asia	India	19	62	25	32	78	90
BCC									
Dokianakis	2000	Caucasian	Greece	3	3	15	6	41	12
Bastiaens	2001	Caucasian	The Netherlands	8	43	63	10	72	75
McGregor	2002	Caucasian	England	0	23	66	5	66	85
Han	2006	Caucasian	USA	23	108	154	45	297	474
Pezeshki	2006	Asian	Iran	10	47	34	86	217	162
Bendesky	2007	Other	Mexico	22	74	108	18	94	126
Almquist	2011	Caucasian	USA	57	295	485	47	274	446
Rizzato	2011	Caucasian	Hungary, Romania, Slovakia	40	186	292	46	178	297

Most of the studies (16 of 20) were conducted in Caucasians. Of the twenty case-control studies, four only focused on melanoma [24,29-31], five on SCC [17,20,22,34,35] and two on BCC [26,33]. Four studies investigated both SCC and BCC [16,21,27,32]. Two explored melanoma, SCC and BCC [18,25]. Two studies investigated non-melanoma skin cancer, without subtype specified [19,28]. And one explored skin cancer, histological subtype not mentioned [23]. The publication year was from 2000 to 2012. The sample sizes ranged from 43 to 1643. All cases were pathologically confirmed. The controls were healthy populations and matched for age, gender and ethnicity. All polymorphisms in the controls were in Hardy-Weinberg equilibrium.

### Meta-analysis Results

**Figure 1:**
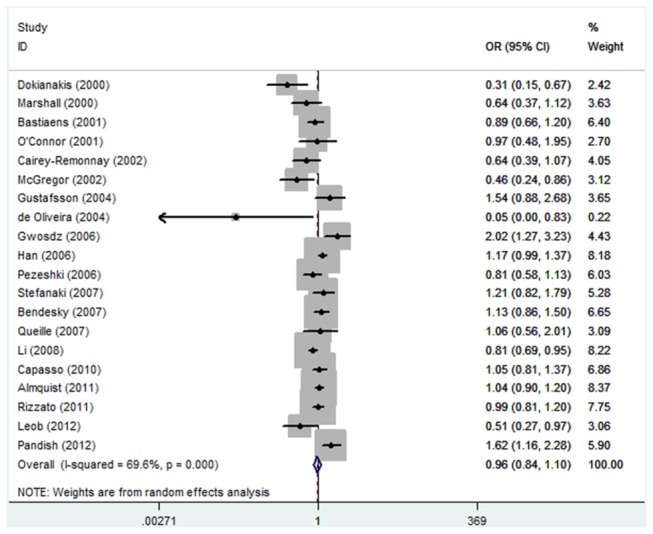
Forest plot of Pro allele versus Arg allele

**Figure 2:**
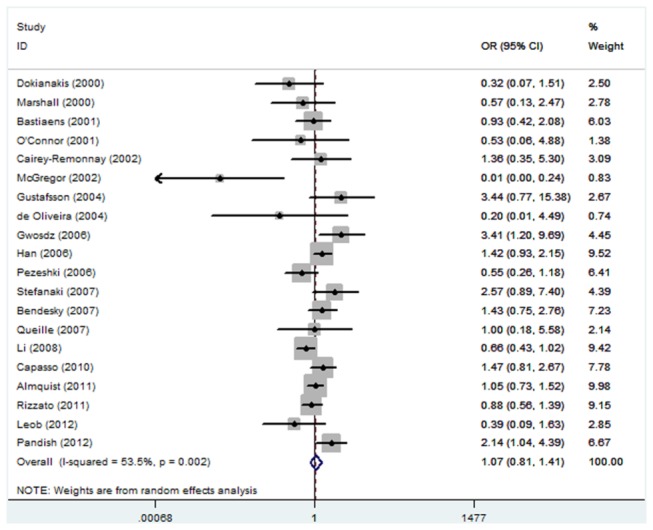
Forest plot of Pro/Pro versus Arg/Arg for all studies.

**Figure 3:**
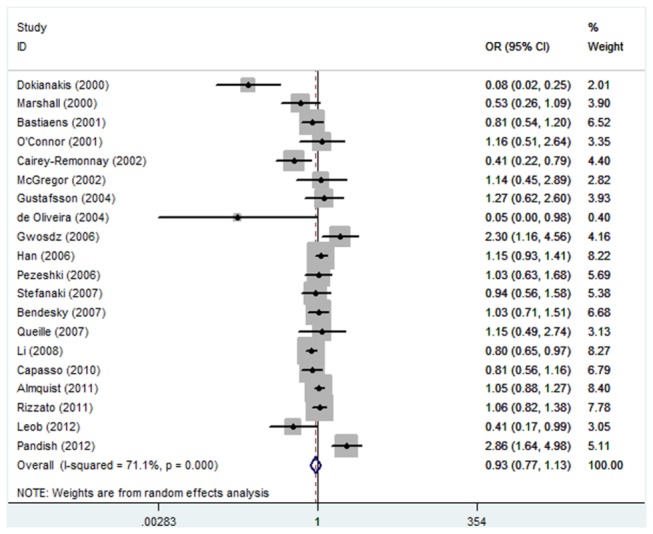
Forest plot of Pro/Arg versus Arg/Arg for all studies.

**Figure 4:**
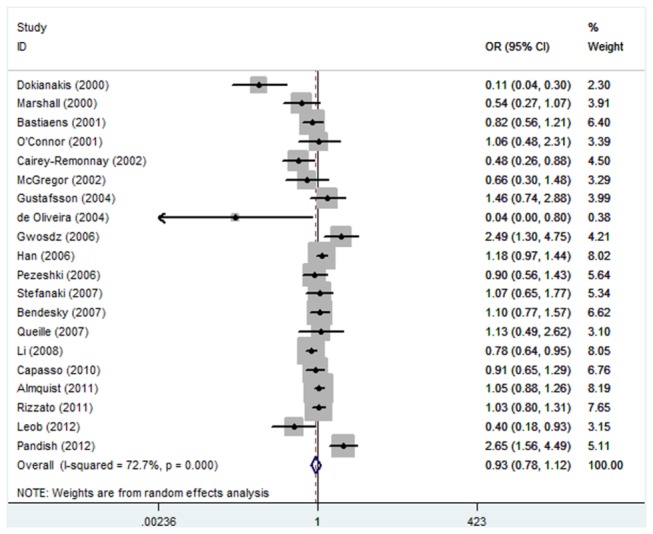
Forest plot of Pro/Pro+ Pro/Arg versus Arg/Arg for all studies.

**Figure 5:**
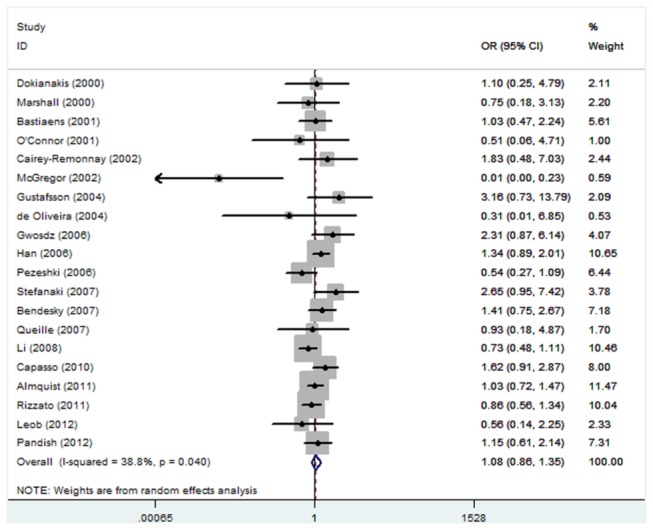
Forest plot of Pro/Pro versus Arg/Arg+ Pro/Arg for all studies.

As shown in Table 2, no significant association between TP53 Arg72Pro polymorphism and the risk of skin cancer was observed in any genetic model and allele contrast (Pro/Pro versus Arg/Arg, odds ratio (OR) =1.07, 95% confidence interval (CI): 0.81-1.41; Pro/Arg versus Arg/Arg, OR=0.93, 95% CI: 0.77-1.13; Pro/Pro + Pro/Arg versus Arg/Arg, OR=0.93, 95% CI: 0.78-1.12; Pro/Pro versus Arg/Arg + Pro/Arg, OR=1.08, 95% CI: 0.86-1.35; Pro allele versus Arg allele, OR=0.96, 95% CI: 0.84-1.10) (Figure 1-5). Power calculations on the pooled frequencies indicated that the statistical powers were all lower than 80% for all the above meta-analyses.

**Table 2:** Main results of meta-analysis for *TP53* Arg72Pro polymorphism and skin cancer risk.

Comparative models	n	Case/Control	OR(95%CI)	P_OR_	I^2^ (%)	P_H_	Model	Power calculation
Total	20	5276/5315						
Pro allele vs. Arg allele			0.96(0.84-1.10)	0.588	62.46	<0.001	random	26.0%
Pro/Pro vs. Arg/Arg			1.07(0.81-1.41)	0.654	40.9	0.002	random	20.2%
Pro/Arg vs. Arg/Arg			0.93(0.77-1.13)	0.468	65.85	<0.001	random	41.2%
Pro/Pro+Pro/Arg vs. Arg/Arg			0.93(0.78-1.12)	0.459	69.52	<0.001	random	44.5%
Pro/Pro vs. Arg/Arg+Pro/Arg			1.08(0.86-1.35)	0.52	31.04	0.04	random	26.9%
Caucasians	16	4822/4385						
Pro allele vs. Arg allele			0.94(0.81-1.09)	0.385	47.54	<0.001	random	44.4%
Pro/Pro vs. Arg/Arg			1.05(0.77-1.43)	0.768	32.41	0.006	random	11.5%
Pro/Arg vs. Arg/Arg			0.88(0.72-1.06)	0.177	46.99	<0.001	random	81.0%
Pro/Pro+Pro/Arg vs. Arg/Arg			0.88(0.73-1.07)	0.203	50.61	<0.001	random	84.4%
Pro/Pro vs. Arg/Arg+Pro/Arg			1.12(0.86-1.46)	0.417	25.99	0.038	random	44.5%
Non-Caucasians	4	454/930						
Pro allele vs. Arg allele			1.06(0.68-1.65)	0.791	77.2	0.004	random	10.8%
Pro/Pro vs. Arg/Arg			1.10(0.52-2.31)	0.801	63.1	0.043	random	8.8%
Pro/Arg vs. Arg/Arg			1.22(0.61-2.42)	0.577	79.6	0.002	random	36.5%
Pro/Pro+Pro/Arg vs. Arg/Arg			1.16(0.59-2.26)	0.671	80.5	0.001	random	24.4%
Pro/Pro vs. Arg/Arg+Pro/Arg			0.95(0.66-1.36)	0.764	37.9	0.185	fixed	6.0%
Melanoma	6	1522/2433						
Pro allele vs. Arg allele			1.10(0.87-1.39)	0.437	75.4	0.001	random	45.2%
Pro/Pro vs. Arg/Arg			1.36(0.82-2.26)	0.232	65.9	0.012	random	67.1%
Pro/Arg vs. Arg/Arg			0.99(0.76-1.28)	0.910	63	0.019	random	5.2%
Pro/Pro+Pro/Arg vs. Arg/Arg			1.05(.079-1.39)	0.745	71.1	0.004	random	11.6%
Pro/Pro vs. Arg/Arg+Pro/Arg			1.33(0.87-2.03)	0.191	54.4	0.052	fixed	62.3%
SCC	11	1455/2643						
Pro allele vs. Arg allele			0.76(0.55-1.06)	0.110	85.7	<0.001	random	100.0%
Pro/Pro vs. Arg/Arg			0.62(0.31-1.25)	0.182	78.2	<0.001	random	98.2%
Pro/Arg vs. Arg/Arg			0.85(0.61-1.19)	0.340	73.8	<0.001	random	80.6%
Pro/Pro+Pro/Arg vs. Arg/Arg			0.75(0.51-1.12)	0.158	83.1	<0.001	random	99.2%
Pro/Pro vs. Arg/Arg+Pro/Arg			0.72(0.42-1.22)	0.219	64.1	0.002	random	83.2%
BCC	8	2159/3179						
Pro allele vs. Arg allele			0.90(0.75-1.08)	0.245	66.3	0.004	random	65.5%
Pro/Pro vs. Arg/Arg			1.01(0.81-1.26)	0.931	30.7	0.183	fixed	5.1%
Pro/Arg vs. Arg/Arg			0.83(0.64-1.08)	0.163	72.5	0.001	random	88.1%
Pro/Pro+Pro/Arg vs. Arg/Arg			0.83(0.64-1.07)	0.140	74.1	<0.001	random	79.1%
Pro/Pro vs. Arg/Arg+Pro/Arg			1.03(0.83-1.28)	0.787	22.9	0.247	fixed	5.7%

Abbreviations: OR, odds ratio; CI, confidence interval; n, number of case-control studies; P_OR_, P value of Z-test; P_H,_ P value for heterogeneity analyses; BCC, basal cell carcinoma; SCC, squamous cell carcinoma.

In the stratified analysis by histological types of skin cancer, there was no evidence of a significant association between codon 72 polymorphism of *TP53* gene and the risk of melanoma, SCC and BCC. Similar results were found in the stratified analysis by ethnicity. Different from other subgroups, power calculations on the SCC gene models were all more than 80%, which revealed adequate sample sizes (Table 2).

### Publication Bias

**Figure 6:**
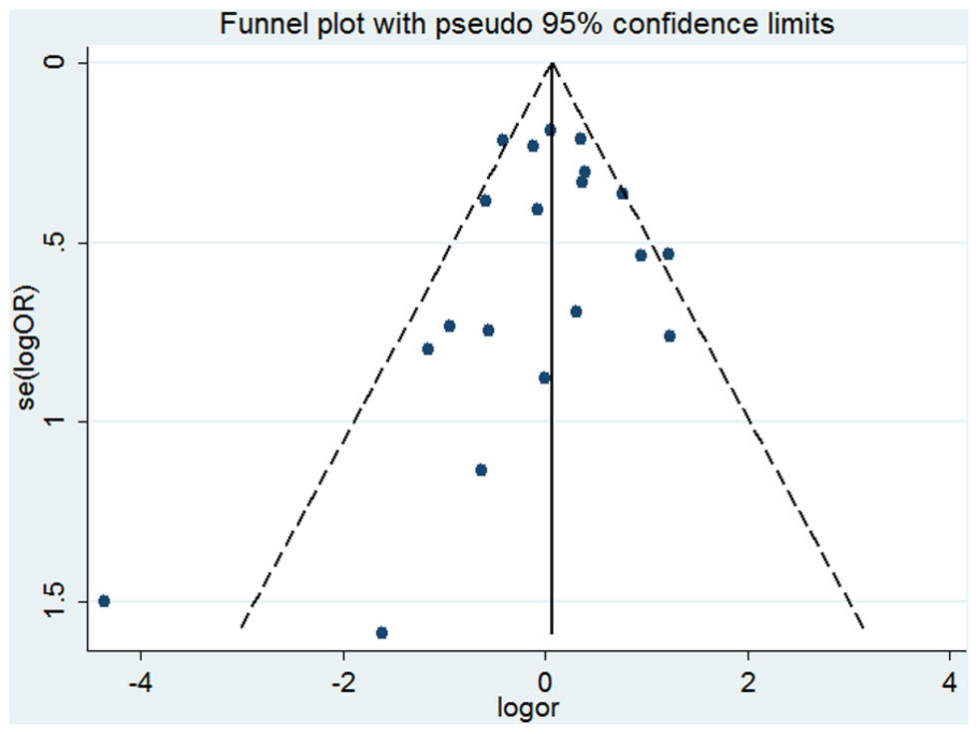
Begg's funnel plot of Pro/Pro versus Arg/Arg for all studies (Begg's Test: P =0.284, Egger's test: P =0.455).

The publication bias was assessed by Begg’s funnel plot and Egger’s test. The shape of the funnel plots was seemed symmetrical and the results of Egger’s test were not significant in all the genetic models (Pro/Pro versus Arg/Arg, Pro/Arg versus Arg/Arg, Pro/Pro + Pro/Arg versus Arg/Arg, Pro/Pro versus Arg/Arg + Pro/Arg, Pro allele versus Arg allele), which indicated no publication bias. Figure 6 shows Begg's funnel plot of overall Pro/Pro versus Arg/Arg. In the stratified analyses by ethnicity and histological types, neither Begg’s funnel plot nor Egger’s test presented any obvious evidence of publication bias (data not shown). These results indicated no publication bias in our meta-analysis.

## Discussion

*TP53* tumor suppressor gene plays an important role in the cell cycle arrest and activation of programmed cell death [8,36]. Mutations of *TP53* gene have been detected in 50% of all human cancers and in almost all skin carcinomas [37]. Studies have proved that inactivation of *TP53* gene involves in the induction of skin cancer by UV radiation [11,12,38]. The most common polymorphism of *TP53* gene locates at codon 72, which is a G-C transversion, causing an amino acid change from arginine (Arg) to proline (Pro) [10]. The functions of the two polymorphic variants of *TP53* gene are different. According to the study conducted by Dumont et al, the Arg72 variant induces cell apoptosis markedly better than the Pro72 variant does [39]. Recently, many studies have explored the association between *TP53* Arg72Pro polymorphism and the susceptibility of skin cancer, but their conclusions are contradictory. Hence, we performed this meta-analysis to further investigate the influence of *TP53* Arg72Pro polymorphism on the development of skin cancer.

The results suggested that no significant association between *TP53* Arg72Pro polymorphism and the risk of skin cancer in any genetic model (Pro/Pro versus Arg/Arg, Pro/Arg versus Arg/Arg, Pro/Pro + Pro/Arg versus Arg/Arg, Pro/Pro versus Arg/Arg + Pro/Arg). In the stratified analysis by ethnicity and histological types of skin cancer, there was no evidence of a significant association, neither. Our results were similar to the meta-analysis conducted by Jiang in 2011 [40].

However, the results of our meta-analysis should be interpreted with caution. Except SCC subgroup, most of the power calculations on the pooled frequencies were lower than 80%, which demonstrated inadequate sample sizes.

This meta-analysis also had some limitations. First, given that only twenty studies were included, publication bias could potentially exit, even though we tried to find as many studies as we could, carefully assessed the literature and used statistical methods to minimize the publication bias, and no statistically significant publication bias was observed in this meta-analysis. Second, in the stratified analyses by ethnicity, most studies were conducted in Caucasians, and information about other ethnicities, such as African, was insufficient. Thus, more studies with larger sample size and high quality, especially for non-Caucasian populations are needed to demonstrate our conclusions in the future. Finally, the case-control study belongs to retrospective research that has methodological deficiencies.

Despite of limitations, this meta-analysis indicated that *TP53* Arg72Pro polymorphism probably had little association with the risk of skin cancer mainly in Caucasians. Nevertheless, it is still necessary to conduct larger size and better- designed studies to explore *TP53* Arg72Pro polymorphism as low statistical powers.

## Supporting Information

Figure S1 **Flow chart of the literature.**
(DOC)

Checklist S1 **PRISMA checklist.**
(DOC)
