# Supplementary figures and images for: Arg72Pro Polymorphism of TP53 Gene and the Risk of Skin Cancer: a Meta-Analysis
Source: PLoS One. 2013 Nov 8;8(11):e79983. doi: 10.1371/journal.pone.0079983 (PMC3832645; doi:10.1371/journal.pone.0079983)

**
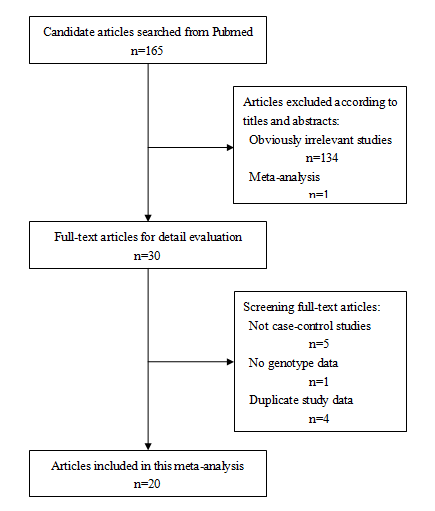
**

**Figure S1** **Flow chart of the literature**

Supplement: Figure S1 — Flow chart of the literature. (DOC) [file pone.0079983.s001.doc]
